# Hypokalemia Duration in the First Year Associated with Subsequent Peritoneal Dialysis-Associated Peritonitis: A Multicenter Retrospective Cohort Study

**DOI:** 10.3390/jcm11247518

**Published:** 2022-12-19

**Authors:** Zhihao Huo, Qianqian Zhuo, Shaoxin Zhong, Fang Wang, Chao Xie, Nirong Gong, Xiaohong Zhong, Zhixiu Yi, Yaozhong Kong, Dehui Liu, Xianrui Dou, Guobao Wang, Jun Ai

**Affiliations:** 1 Division of Nephrology, Nanfang Hospital, Southern Medical University, Guangzhou 510515, China; 2 National Clinical Research Center for Kidney Disease, State Key Laboratory of Organ Failure Research, Guangdong Provincial Institute of Nephrology, Guangdong Provincial Key Laboratory of Renal Failure Research, Guangzhou 510515, China; 3 Department of Nephrology, Shunde Hospital, Southern Medical University, Foshan 528308, China; 4 Department of Nephrology, Nanfang Hospital, Ganzhou (Ganzhou People’s Hospital), Ganzhou 341000, China; 5 Nephrology Department, The First People’s Hospital of Foshan, Foshan 528000, China

**Keywords:** hypokalemia duration, severe hypokalemia, peritoneal dialysis-associated peritonitis

## Abstract

Background: The association of hypokalemia (LK) with peritoneal dialysis-associated peritonitis (PDAP) risk remains uncertain. Here, we calculated LK duration in the first PD year and evaluated its association with PDAP. Methods: A multicenter, retrospective, incident cohort study of 1633 participants was conducted from January 2008 to October 2020 in China. The duration of LK and severe hypokalemia (SLK) was calculated as the total number of months that a patient’s serum potassium (SK) level was less than 3.5 or 3.0 mEq/L during the first PD year. The study outcome was the risk of subsequent PDAP started in the second year and later. Cox proportional hazards models and competing risk models were used to assess the association. Results: The subsequent PDAP occurred in 420 (25.7%) participants during a median of 28 months of follow-up. Overall, LK duration in the first year was positively associated with a subsequent PDAP risk (per 3-month increments, adjusted HR, 1.13; 95%CI: 1.05–1.23). After categorization, patients with LK duration longer than 6 months had the highest adjusted HR of 1.53 (*p* = 0.005 vs. those without LK) for subsequent PDAP risk. A similar trend was also found for SLK duration. In a competing risk model, a similar trend was also observed. None of the variables, including demographic and PD characteristics, diabetes history, and several clinical measurements, significantly modified this association. The causative organisms of PDAP were similar to those previously reported. Conclusions: PD patients with longer LK duration in the first year had a higher subsequent PDAP risk.

## 1. Introduction

Peritoneal dialysis (PD)-associated peritonitis (PDAP) is a serious complication among PD patients, with a prevalence of 0.26–0.40 episodes per person-year [1], resulting in peritoneal membrane alterations, peritoneal adhesions, hemodialysis transfer, and even death [2,3,4,5,6]. As such, it is of great clinical significance to identify more modifiable related factors for the prevention of PDAP. Apart from those conventional factors including age, gender, high body mass index (BMI), smoking, diabetes history, and malnutrition, hypokalemia (LK) was also reported to be an important factor causing PDAP [7,8,9,10,11].

Hypokalemia (defined as serum potassium [SK] less than 3.5 mEq/L) is far more common in patients undergoing peritoneal dialysis (PD) than in those subjected to hemodialysis, with a frequency ranging from 10% to 58.6% [12,13,14] and was reported to be associated with a series of clinical outcomes, such as cardiovascular events, all-cause mortality, and PDAP [12,14,15]. Over the decades, the association of LK with PDAP has remained inconclusive. Some studies reported a positive association [14,15], while Fan et al. [16] and Goncalves et al. [17] denied it. Interestingly, two studies with positive associations used repeated SK values after PD initiation to evaluate LK [14,15], whereas Fan’s study [16] with negative result only used baseline SK values. To our knowledge, patients have a higher prevalence of LK after PD therapy; in addition, the serum potassium level changes dynamically over time [18,19]. Hence, those data based on repeated SK measurements after PD initiation seemed to be more reasonable. Furthermore, the studies mentioned above mainly focused on one time point measurement [16,20] or on time-averaged values [12,15,17] to evaluate LK, which might overlook the serial alterations and detailed dynamic changes of SK, especially under diet control and dialysis treatment.

Recently, Davies et al. [21] calculated LK duration during the 4 months before PD treatment using data from the Peritoneal Dialysis Outcomes and Practice Patterns Study (PDOPPS) and confirmed the higher hazard ratio (HR) of first PDAP occurrence in patients with longer LK duration. Admittedly, LK duration might partially represent the dynamic changes of SK. Anyhow, data on the correlation of LK duration after dialysis with PDAP remain limited. Thus, we conducted a multicenter cohort study among PD patients, aiming to explore the association between LK duration in the first year and the risk of subsequent PDAP started in the second year and later and examine their possible effect modifiers.

## 2. Materials and Methods

### 2.1. Design and Participants

**Figure 1:**
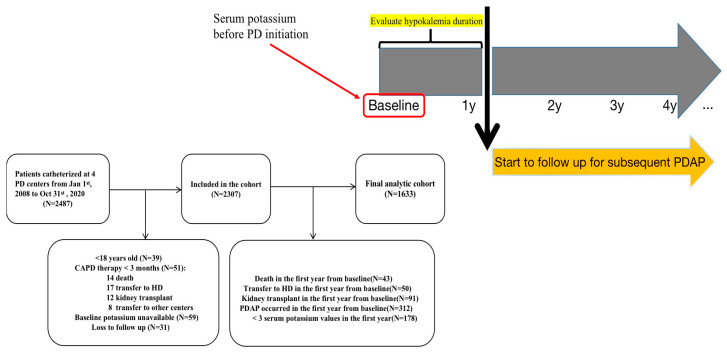
Flowchart of the participants in the current analysis. Notes: Baseline measurement of serum potassium was collected before PD initiation. Hypokalemia duration was calculated in the first PD year. The endpoint of the study was subsequent PDAP, which was followed up starting from the second year. Abbreviations: PD, peritoneal dialysis; CAPD, continuous ambulatory peritoneal dialysis; HD, hemodialysis; PDAP, peritoneal dialysis-associated peritonitis.

The present study was a retrospective, multicenter, incident cohort study conducted in 4 dialysis centers of southern China (including Nanfang Hospital, Ganzhou People’s Hospital, the First People’s Hospital of Foshan, and Shunde Hospital, Southern Medical University). The initial cohort consisted of 2487 ESRD patients who had started on continuous ambulatory peritoneal dialysis (CAPD) between 1 January 2008, and 31 October 2020. The following patients were excluded: age <18 years (*n* = 39), CAPD therapy for less than 3 months (*n* = 51), baseline SK unavailable (*n* = 59), loss to follow-up (*n* = 31), PD withdrawal including death, transfer to HD and kidney transplantation in the first year from baseline (*n* = 184), PDAP occurring in the first year from baseline (*n* = 312), and less than 3 serum potassium measurements in the first year (*n* = 178). Hence, a total of 1633 participants were included in the final analysis (Figure 1). This study was approved by the Research Ethics Committee of Nanfang Hospital, Guangzhou, China (ethics number NFEC-201909-K17).

### 2.2. Data Collection and Definitions

Baseline (before PD initiation) demographic characteristics (age, gender, education level, and BMI), lifestyle behaviors (smoking and alcohol drinking), etiology of ESRD, initiation of PD, and comorbid conditions (diabetic mellitus, cardiovascular disease [CVD] including congestive heart failure, coronary artery disease, peripheral vascular disease, and cerebrovascular disease) were collected from the medical records. Data of dialysate glucose concentration (GLUC), 24 h PD ultrafiltration (UF) volume, 24 h urine volume (UV), weekly total Kt/V were recorded at baseline (1 month after PD initiation), every 3 months for the first two years after PD initiation, and every 6 months starting in the third year. Similarly, systolic blood pressure (SBP), diastolic blood pressure (DBP), measurements of serum biochemical parameters including SK, serum levels of creatinine, albumin (ALB), calcium, phosphorus, intact parathyroid hormone (iPTH), fasting glucose, blood hemoglobin (HGB), medications including renin–angiotensin system inhibitors (RASi) and diuretic were collected on every regular follow-up visit at baseline (before PD initiation, 0 month) and at fixed times (every 3 months for the first two years and subsequently at 6 month intervals). All biochemical parameters were measured by using standardized and automated methods in the involved 4 centers.

Dialysate GLUC (%) = Σ (glucose concentration × input volume)/total input volume. BMI was calculated as weight (kg) divided by height in meters squared (m^2^). MAP was approximated as (⅓ · SBP + ⅔ · DBP). Conventional weekly total Kt/V was calculated by standard methods [22]. Residual renal function (RRF) loss was defined as 24 h UV < 100 mL.

### 2.3. Covariates

LK was defined as SK < 3.5 mEq/L, and severe hypokalemia (SLK) was defined as SK < 3.0 mEq/L [23]. LK and SLK duration were evaluated based on the SK values during the first PD year, respectively. Accordingly, LK duration (months) in the first year = Σ (PD vintage when SK concentration was less than 3.5 mEq/L—previous nearest PD vintage when SK concentration was not less than 3.5 mEq/L). It should be noted that LK duration was measured as 12 months if the SK concentrations were less than 3.5 mEq/L at all follow-up points, and as 0 month if the SK measurements were not less than 3.5 mEq/L at all time points. In addition, when there existed a missing value at a follow-up point, we evaluated whether it was less than 3.5 mEq/L at its nearest previous and subsequent follow-up points. Only when both values before and after this follow-up point were less than 3.5 mEq/L, this missing value was estimated as LK, otherwise it was regarded as without LK. Similarly, SLK duration (months) in the first year = Σ (PD vintage when SK concentration was less than 3.0 mEq/L—previous nearest PD vintage when SK concentration was not less than 3.0 mEq/L).

### 2.4. Study Outcome

The study outcome was subsequent PDAP, which was diagnosed according to the International Society for Peritoneal Dialysis (ISPD) recommendations [2], when at least two of the following criteria were met: (1) clinical features consistent with peritonitis, that is, abdominal pain and/or cloudy dialysis effluent; (2) dialysis effluent white cell count >100/µL or >0.1 × 10^9^/L (after a dwell time of at least 2 h), with >50% polymorphonuclear leukocytes; (3) positive dialysis effluent culture. The follow-up time for subsequent PDAP started in the second year (Figure 1).

All enrolled participants were followed until death or the end of the study (31 October 2021). Causes of censoring were patients who transferred to HD, received kidney transplantation, lost to follow-up, or transferred to other dialysis centers.

### 2.5. Statistical Analysis

Means ± standard deviations (SDs) or medians [interquartile range (IQR)] for continuous variables and proportions for categorical variables were calculated. The differences in population characteristics by LK duration in the first PD year were compared using ANOVA tests or chi-square tests, accordingly. Cumulative hazards of subsequent PDAP by LK duration categories were estimated using the Kaplan–Meier method, and group differences were compared by log-rank tests.

The associations between LK and SLK duration (continuous and categories) in the first PD year and the risk of subsequent PDAP started in the second year and later were estimated by Cox proportional hazards models (HR and 95%confidence interval [95%CI]) without and with adjustments for age, gender, BMI, education level, smoking, diabetes, CVD history, RASi medications, RRF loss (with or without, in the first year), baseline SK, and the mean values of dialysate GLUC, total weekly Kt/V score, serum ALB, creatinine and phosphorus in the first PD year. To accurately assess the risk of subsequent PDAP, the events including death, kidney transplantation, and HD transfer were considered as competing risk factors. Thus, Fine–Gray competing-risk regression models were used to estimate sub-distribution hazard ratio (sHR) by adjusting the same confounders in the Cox proportional hazards models above. Moreover, possible modifications of the association between LK duration in the first year and risk of subsequent PDAP started in the second year and later were evaluated by stratified analyses, and their interactions were assessed. Organisms between the LK group and the group without LK were compared by the chi-square tests.

A two-tailed *p* value < 0.05 was considered statistically significant in all analyses. The analyses were performed using the statistical packages R (The R Foundation; http://www.R-project.org; version 4.2.0) and EmpowerStats (www.empowerstats.net, accessed on 23 August 2022, X&Y solutions, Inc., Boston, MA, USA).

## 3. Results

### 3.1. Participant Characteristics

As shown in the flowchart (Figure 1), a total of 1633 participants were included in the final analytic cohort. Among them, 54.0% were male, and the average age was 46.6 years (SD, 13.8). The incidence of LK and SLK (at least one episode in the first PD year) was 46.9% and 15.6%, respectively. The median LK and SLK duration within the first year was 4.5 (IQR, 3–9) and 3 (IQR, 3–6) months. The detailed participant characteristics by LK duration in the first PD year are listed in Table 1. Patients with longer LK duration were more likely to have lower BMI, baseline MAP, baseline serum phosphorus, and lower frequency in diuretic and RASi use.

### 3.2. Associations of LK and SLK Duration with Subsequent PDAP Risk

**Figure 2:**
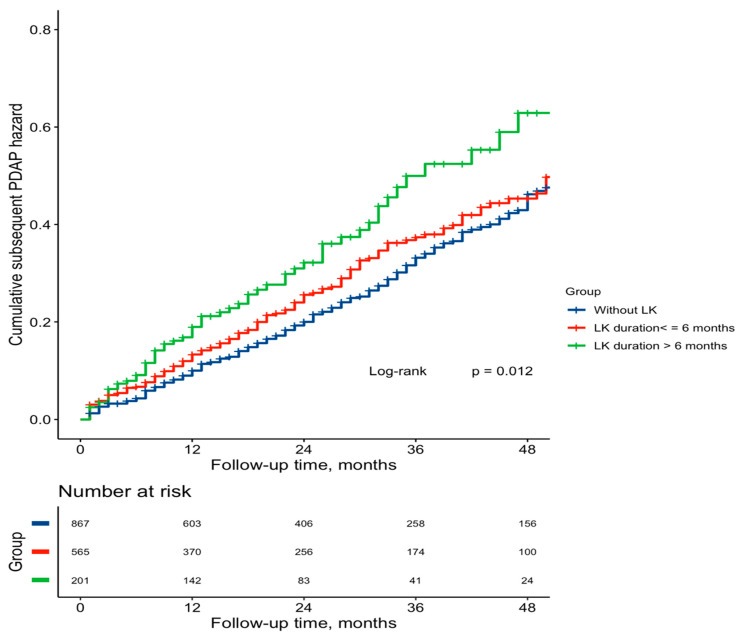
Kaplan–Meier curves for the cumulative hazard of subsequent PDAP. Notes: The follow-up time for subsequent PDAP started in the second year. Group differences were compared by log-rank tests. Abbreviations: PDAP, peritoneal dialysis-associated peritonitis; LK, hypokalemia.

During the median follow-up of 28 months (IQR, 12–47 months), 420 (25.7%) patients were identified with subsequent PDAP started in the second year and later (0.15 episodes per patient-year). The subsequent PDAP rate was 0.17 episodes per patient-year in hypokalemic patients, which was higher than that in the non-hypokalemic group (0.14 episodes per patient-year). As shown in Table 2, LK duration in the first PD year was positively associated with a subsequent PDAP risk (per 3-month increments: adjusted HR, 1.13; 95%CI, 1.05–1.23, *p* = 0.002). Consistently, compared to patients without LK (LK duration = 0), the subsequent PDAP risk was significantly higher, with HR of 1.53 (95%CI, 1.14–2.06, *p* = 0.005), in those with LK duration for more than 6 months (Table 2). In the competing risk model, LK duration in the first PD year was still positively associated with subsequent PDAP risk when considering death, kidney transplantation, and HD transfer as competing events. A similar trend was found for SLK duration (per 3-month increments, adjusted HR, 1.19; 95%CI: 1.02–1.38, *p* = 0.027) (Table 2). Additionally, the Kaplan–Meier curve of the cumulative event rate of subsequent PDAP within each LK duration strata is shown in Figure 2.

### 3.3. Stratified Analyses

**Figure 3:**
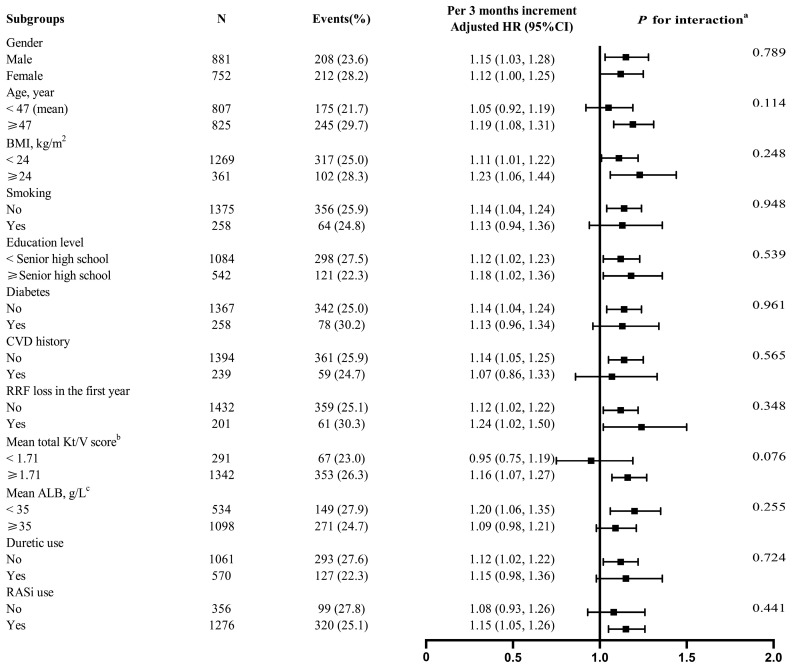
Association of LK duration in the first PD Year with subsequent PDAP risk in various subgroups. Notes: ^a^ If not stratified, adjusted for age, gender, BMI, education level, smoking, diabetes, CVD history, RASi medications, RRF loss (with or without, in the first year), baseline potassium, and the mean values of dialysate GLUC, total weekly Kt/V score, serum albumin, serum creatinine and serum phosphorus in the first PD year. ^b,c^ The mean values of total Kt/V score and ALB in the first PD year were used. Abbreviations: LK, hypokalemia; PD, peritoneal dialysis; PDAP, peritoneal dialysis-associated peritonitis; HR, hazards ratio; CI, confidence interval; BMI, body mass index; CVD cardiovascular disease; RRF, residual renal function; ALB, albumin; RASi, renin–angiotensin system inhibitor; GLUC, dialysate glucose concentration.

To better understand other possible influencing factors in the relationship between LK duration in the first PD year and risk of subsequent PDAP started in the second year and later, further exploratory subgroup analyses were conducted. None of the variables, including gender (male vs. female), age (<47 vs. ≥47 years), BMI (<24 vs. ≥24 kg/m^2^), smoking (no vs. yes), education level (<senior high school vs. ≥senior high school), diabetes (no vs. yes), CVD history (no vs. yes), RRF loss in the first year (no vs. yes), mean total Kt/V score (<1.71 vs. ≥1.71), mean ALB (<35 vs. ≥35 g/L), diuretic use (no vs. yes), as well as RASi use(no vs. yes) significantly modified the association between LK duration in the first year and subsequent PDAP risk (all *p*-interactions > 0.05) (Figure 3).

### 3.4. Causative Organisms of the Subsequent PDAP

**Figure 4:**
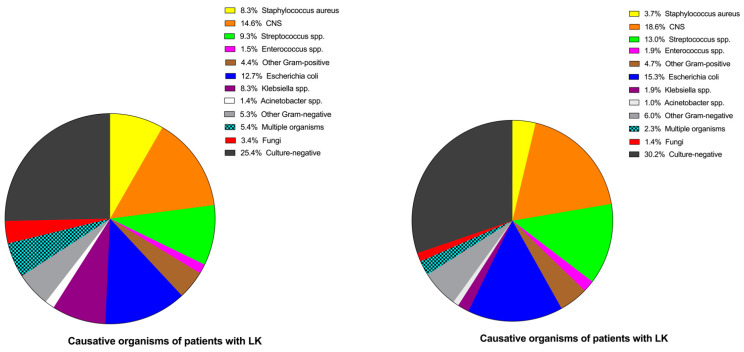
Distribution of causative organisms in PD patients with and without LK. Notes: Comparisons of causative organisms in patients with LK (**left**) and without LK (**right**) in the first PD year. LK here was defined as at least one episode in the first PD year. CNS includes S epidermidis, S hominis, S capitis, S warneri, and S hemolyticus. Abbreviations: PD, peritoneal dialysis; LK, hypokalemia; CNS, coagulase-negative Staphylococcus; spp., species.

The causative organisms after patient stratification according to the presence of LK are presented in Table 3 and Figure 4. Gram-positive bacteria were the predominant pathogen in the whole cohort. Of the 420 episodes of subsequent PDAP, 168 (40.0%) were due to Gram-positive organisms, 109 (25.9%) episodes were caused by Gram-negative germs, 10 (2.4%) episodes were due to fungi, and 16 (3.8%) to multiple organisms. Culture-negative results were reported in 117 (27.9%) cases. The most frequent of Gram-positive organisms was coagulase-negative Staphylococcus (CNS) (16.7%), and the most encountered Gram-negative germ was *Escherichia coli* (59 cases), accounting for 14.0% of the subsequent PDAP episodes. Compared to patients with LK, those without LK presented more than twice the incidence of subsequent *staphylococcus aureus*-associated PDAP (8.3% vs. 3.7%, *p* = 0.048). There was no significant difference in the incidence of *Enterobacteriaceae*-associated PDAP between the LK group and the group without LK.

## 4. Discussion

In this multicenter cohort study, we identified that LK duration in the first PD year was positively associated with subsequent PDAP started in the second year and later, with a median follow-up of 28 months, among Chinese incident PD patients, which provides clinical evidence of the need of hypokalemia management in the PD population.

Previous studies concerning the relationship between LK and PDAP are controversial. A retrospective cohort study conducted by Chuang et al. [14] showed that hypokalemic patients had a higher risk of PDAP compared to those without LK during the whole follow-up period. In the Brazilian PD Multicenter Study (BRAZPD), time-averaged LK was associated with a higher risk for PDAP [15]. In contrast, Fan’s study [16] did not find an association between LK and PDAP by using baseline LK. It is noteworthy that the discrepancy of these above studies might depend on how LK was defined. Specifically, both Chuang’s [14] and BRAZPD’s [15] studies used repeated serum potassium values after PD initiation to define LK, whereas the other study [16] defined LK by using one time point measurement. Notably, our prior study reported that the serum potassium level decreased rapidly from baseline and changed dynamically over time under dialysis therapy and diet control [18]. As such, it seems more reasonable to use post-dialysis repeated data to define LK. Furthermore, using baseline or time-averaged values might lead to overlooking the serial alterations and detailed dynamic changes in serum potassium among PD patients. In our study, LK duration was calculated based on measurements after dialysis in the first PD year, which might reflect the dynamic changes of serum potassium. Patients with an increment in LK every 3 months displayed 13% higher adjusted subsequent PDAP hazards. Furthermore, patients with an even longer LK duration (more than 6 months) had the highest subsequent PDAP risk. Consistently, the positive association of LK duration with subsequent PDAP risk remained stable after considering competing events including death, kidney transplantation, and HD transfer. These data confirmed the association between hypokalemia and PDAP risk and highlight the clinical importance of hypokalemia monitoring.

To our knowledge, the degree of LK might be associated with a differential PDAP risk [12,15,18]. Patients with SLK might suffer from even greater appetite reduction and gastrointestinal motility disorders, leading to PDAP. Our findings showed that patients with subsequent PDAP that started in the second year and later presented a higher risk of 19% for every 3-month increase in SLK duration during the first year. After categorization, the PD patients with the longest SLK duration presented the highest HR of PDAP, which was 117% higher than that of the patients without SLK. As such, the degree of hypokalemia should also be sufficiently evaluated in clinical practice.

Over the decades, most studies have focused on the first occurrence of PDAP [11,14,15,17,20,21]. A randomized controlled trial [11] demonstrated that potassium supplementation can significantly prolong the time to the first PDAP episode, which strongly suggests a causal relationship between LK and PDAP. To better understand the association between LK and subsequent PDAP, the present study firstly excluded patients with PDAP in the first PD year at enrollment and then examined the relationship between LK duration in the first year and subsequent PDAP that started in the second year and later. Even though a retrospective cohort study cannot prove causality, our design could confirm the chronological relationship, which partially indicated that LK might be a causal factor for PDAP. However, further perspective cohort studies and RCTs are warranted to verify this conclusion.

The mechanism of LK leading to a higher risk of PDAP is incompletely understood. One possible explanation of our findings might be that LK was reported to be associated with dysmotility of the small intestine, which might lead to intestinal dilatation and gut wall ischemia and thus enhance intra-abdominal pressure, bacterial translocation, and, ultimately, the susceptibility to PDAP [14,24]. Given the complex relationship between LK and PDAP, more specific mechanistic studies are required.

In general, loop diuretics are less commonly used in PD patients because of renal severe injury and anuria. However, spironolactone, a kind of aldosterone antagonist [25], is more frequently prescribed to treat hypokalemia in this population. In our cohort, the diuretics used were mainly spironolactone, which partly explains why patients using fewer diuretics might be more prone to hypokalemia.

In our multicenter cohort, we found a subsequent PDAP rate of 0.15 episodes per patient-year. CNS in Gram-positive and Escherichia coli in Gram-negative bacteria were the main organisms in our cohort, which is consistent with other studies [26,27,28]. We observed that patients without LK had more than twice the incidence of subsequent staphylococcus aureus-associated PDAP (8.3% vs. 3.7%), compared to hypokalemic PD patients. According to the finding of Chuang et al. [14], hypokalemic patients were more likely to acquire *Enterobacteriaceae*-associated peritonitis. Unfortunately, our study did not find an association between them. The reasons might be as follows: firstly, the culture-negative proportion was higher than that indicated by the ISPD (lower than 15%) due to patients’ use of antibiotic drugs in local hospitals before their transfer to our four dialysis centers, which might partially be a confounding factor that weakened the role of LK in PDAP. Secondly, patients with PDAP within the first PD year were excluded, which might have caused a selection bias. Thirdly, hypokalemic patients were more prone to fatigue, thus adhered less to sterile practice recommendations, resulting in more frequent touch contamination. Interestingly, the PDOPPS study [21] also found no association between mean potassium concentration <3.5 mEq/L during the 4 months before enrollment and PDAP risk caused by enteric or Gram-negative organisms, but a tendency toward a higher risk of Gram-positive-associated peritonitis (adjusted HR 1.14, 95%CI: 0.91–1.43).

There are several limitations in our study. First, our data set did not include the potassium dietary intake in relation to SK and the use of potassium supplementation, which could be a confounding factor that weakened our results if patients used it. However, the potassium measurements in our cohort reflected the potassium status of the patients and were not recorded after potassium supplementation, if any. Secondly, PD patients who were excluded during the first 12 months after PD initiation might weaken the generalizability of the cohort. Thirdly, the cohort study was conducted in southern China; therefore, the generalization of our results to other ethnicities and populations still needs further research. Finally, although adjusting for these covariates, we cannot exclude the possibility of residual confounding from other unmeasured or unrecorded risk factors.

## 5. Conclusions

In summary, our results provide insights into the positive association between LK duration in the first year after PD initiation and subsequent PDAP risk. Our findings suggest the clinical importance of hypokalemia monitoring in PDAP patients. Further larger studies are warranted to confirm these findings.

## Figures and Tables

**Table 1:** Characteristics of the study participants by LK duration in the first PD year.

Characteristic		LK Duration in the First PD Year, Months		
	Total	0	≤6	>6
No. of participants	1633	867	565	201
Age, years	46.6 ± 13.8	45.8 ± 13.1	47.2 ± 14.3	47.9 ± 15.2
Male, No. (%)	881 (54.0)	498 (57.4)	282 (49.9)	101 (50.3)
BMI, kg/m^2^	21.8 ± 3.3	22.1 ± 3.1	21.5 ± 3.3	21.4 ± 3.5
Smoking, No. (%)	258 (15.8)	144 (16.6)	85 (15.0)	29 (14.4)
Alcohol drinking, No. (%)	133 (8.1)	78 (9.0)	41 (7.3)	14 (7.0)
Education level, No. (%)				
Junior high school and below	1084 (66.7)	560 (65.0)	389 (69.1)	135 (67.2)
Senior high school and above	542 (33.3)	302 (35.0)	174 (30.9)	66 (32.8)
DKD to ESRD, No. (%)	258 (15.9)	138 (16.0)	86 (15.3)	34 (17.0)
CVD history, No. (%)	239 (14.6)	121 (14.0)	92 (16.3)	26 (12.9)
PD vintage, months	28 (12–47)	27 (12–48)	29 (10–48)	27 (13–42)
Baseline MAP, mmHg	104.9 ± 14.2	105.9 ± 14.5	103.9 ± 14.1	103.0 ± 13.1
eGFR, ml/min/1.73 m^2^	5.0 ± 2.2	5.1 ± 2.2	5.0 ± 2.1	5.1 ± 2.5
Baseline laboratory results				
Blood HGB, g/L	82.9 ± 19.3	82.9 ± 19.4	83.5 ± 19.9	81.1 ± 17.4
Serum creatinine, μmol/L	980.8 ± 365.9	993.7 ± 376.6	963.1 ± 327.9	974.5 ± 423.3
Serum ALB, g/L	36.5 ± 5.4	36.5 ± 5.4	36.5 ± 5.4	36.3 ± 5.3
Serum potassium, mmol/L	4.6 ± 0.8	4.7 ± 0.8	4.5 ± 0.8	4.3 ± 0.8
Serum calcium, mmol/L	2.0 ± 0.3	2.0 ± 0.3	2.0 ± 0.3	2.0 ± 0.3
Serum iPTH, pg/mL	303.6 (157.0–488.0)	299.3 (157.8–471.5)	324.5 (154.5–497.5)	331.2 (156.5–498.2)
Serum phosphorus, mmol/L	2.1 ± 0.7	2.2 ± 0.7	2.1 ± 0.7	2.0 ± 0.7
Fasting glucose, mmol/L	5.7 ± 2.2	5.8 ± 2.3	5.6 ± 2.1	5.6 ± 2.0
Baseline PD characteristics				
Dialysate GLUC, %	1.6 ± 0.2	1.6 ± 0.2	1.6 ± 0.2	1.6 ± 0.1
UF volume, ml/24 h	320 (200–500)	300 (200–500)	350 (200–500)	325 (200–500)
Urine volume, ml/24 h	1000 (600–1200)	1000 (650–1300)	900 (600–1200)	1000 (550–1200)
Mean values of total Kt/V score ^a^	2.2 ± 0.6	2.2 ± 0.6	2.2 ± 0.6	2.2 ± 0.6
Medication use in the first year, No. (%)				
Diuretic	570 (35.0)	329 (38.0)	197 (34.9)	44 (21.9)
RASi	1276 (78.2)	708 (81.8)	424 (75.0)	144 (71.6)

Notes: Continuous variables are presented as mean ± SD or IQR (25th, 75th), categorical variables are presented as No. (%). ^a^ Mean values of total Kt/V score in the first PD year were used. Abbreviations: LK, hypokalemia; PD, peritoneal dialysis; BMI, body mass index; DKD, diabetic kidney disease; ESRD, end-stage renal disease; CVD, cardiovascular disease; MAP, mean arterial pressure; eGFR, estimated glomerular filtration rate; HGB, hemoglobin; iPTH, intact parathyroid hormone; GLUC, glucose concentration; UF, ultrafiltration; RASi: renin–angiotensin system inhibitor.

**Table 2:** Association of LK and SLK duration in the first PD year with subsequent PDAP.

Subsequent PDAP	N	Events, N (%)	Crude Model ^a^		Adjusted Model ^b^	
			HR (95%CI)	*p*-Value	HR (95%CI)	*p*-Value
Cox model						
LK duration						
Continuous, per 3 months	1633	420 (25.7)	1.13 (1.05, 1.21)	0.002	1.13 (1.05, 1.23)	0.002
Categories						
0 month	867	205 (23.6)	Ref.		Ref.	
≤6 months	565	150 (26.5)	1.16 (0.94, 1.43)	0.165	1.18 (0.94, 1.48)	0.146
>6 months	201	65 (32.3)	1.51 (1.14, 2.00)	0.004	1.53 (1.14, 2.06)	0.005
SLK duration						
Continuous, per 3 months	1633	420 (25.7)	1.23 (1.06, 1.42)	0.006	1.19 (1.02, 1.38)	0.027
Categories						
0 month	1378	345 (25.0)	Ref.		Ref.	
≤6 months	234	65 (27.8)	1.18 (0.91, 1.54)	0.220	1.12 (0.85, 1.49)	0.414
>6 months	21	10 (47.6)	2.54 (1.35, 4.76)	0.004	2.17 (1.14, 4.14)	0.019
Competing risk model						
LK duration						
Continuous, per 3 months	1633	420 (25.7)	1.12 (1.06, 1.19)	0.002	1.13 (1.06, 1.21)	0.003
Categories						
0 month	867	205 (23.6)	Ref.		Ref.	
≤6 months	565	150 (26.5)	1.22 (1.03, 1.46)	0.056	1.21 (1.00, 1.47)	0.095
>6 months	201	65 (32.3)	1.47 (1.16, 1.86)	0.007	1.51 (1.18, 1.94)	0.007
SLK duration						
Continuous, per 3 months	1633	420 (25.7)	1.22 (1.09, 1.37)	0.004	1.18 (1.03, 1.34)	0.038
Categories						
0 month	1378	345 (25.0)	Ref.		Ref.	
≤6 months	234	65 (27.8)	1.16 (0.93, 1.44)	0.270	1.06 (0.84, 1.35)	0.680
>6 months	21	10 (47.6)	2.64 (1.61, 4.32)	0.001	2.28 (1.33, 3.89)	0.011

Notes: The follow-up time for subsequent PDAP started in the second year. ^a^ Crude Model: We did not adjust other covariates. ^b^ Adjusted Model: adjusted for age, gender, BMI, education level, smoking, diabetes, CVD history, RASi medications, RRF loss (with or without, in the first year), baseline potassium, and the mean values of dialysate GLUC, total weekly Kt/V score, serum albumin, serum creatinine and serum phosphorus in the first PD year. In the competing risk model, death, kidney transplantation, and transfer to HD were considered as competing events. And sHR was used in competing risk model. Abbreviations: LK, hypokalemia; SLK, severe hypokalemia; PD, peritoneal dialysis; PDAP, peritoneal dialysis-associated peritonitis; HR, hazards ratio; CI, confidence interval; Ref, reference; sHR, sub-distribution hazard ratio; BMI, body mass index; RASi: renin–angiotensin system inhibitor; RRF, residual renal function; GLUC, dialysate glucose concentration.

**Table 3:** Comparisons of causative organisms in patients with and without LK in the first PD year.

Organisms	Overall	Without LK	With LK	*p*-Value
No. of PDAP	420	205	215	
Gram-positive	168 (40.0)	78 (38.1)	90 (41.9)	0.425
*Staphylococcus aureus*	25 (6.0)	17 (8.3)	8 (3.7)	0.048
CNS ^a^	70 (16.7)	30 (14.6)	40 (18.6)	0.275
*Streptococcus* spp.	47 (11.2)	19 (9.3)	28 (13.0)	0.222
*Enterococcus* spp.	7 (1.7)	3 (1.5)	4 (1.9)	0.949
Others	19 (4.4)	9 (4.4)	10 (4.7)	0.898
Gram-negative	109 (25.9)	57 (27.7)	52 (24.2)	0.398
*Escherichia coli*	59 (14.0)	26 (12.7)	33 (15.3)	0.432
*Klebsiella* spp.	21 (5.0)	17 (8.3)	4 (1.9)	0.003
*Acinetobacter* spp.	5 (1.2)	3 (1.4)	2 (1.0)	0.957
Others	24 (5.7)	11 (5.3)	13 (6.0)	0.764
*Enterobacteriaceae*	80 (19.0)	43 (21.0)	37 (17.2)	0.326
Multiple organisms	16 (3.8)	11 (5.4)	5 (2.3)	0.104
Fungi	10 (2.4)	7 (3.4)	3 (1.4)	0.300
Culture-negative	117 (27.9)	52 (25.4)	65 (30.2)	0.266

Notes: Variables are shown as No. of episodes (%). ^a^ Coagulase-negative staphylococcus includes S epidermidis, S hominis, S capitis, S warneri, and S hemolyticus. Abbreviations: LK, hypokalemia; PD, peritoneal dialysis; PDAP, peritoneal dialysis-associated peritonitis; CNS, coagulase-negative Staphylococcus; spp., species.

## Data Availability

The data analyzed during the current study are available from the corresponding author on reasonable request.
